# Medical Matters in Prussia

**Published:** 1845-06

**Authors:** John H. Dix

**Affiliations:** Berlin


### MEDICAL MATTERS IN PRUSSIA.

*Berlin, Dec.* 15*th*, 1844.

To the Editor of the Boston Medical and Surgical Journal.

*Dear Sir*,—In sending you a few lines from this place, I am not quite certain whether it is a compliance with a request from yourself, or merely a gratification of a desire not to be wholly forgotten by yourself and some of your readers.

I have been here nearly a month, and heard several of the professors at the medical school, which ranks now as the second in Germany, although none of them are superior as lecturers to many in America, and the graces of oratory are as foreign to the profession here as elsewhere. One is a little surprised to see in nearly all the lecture rooms of a Prussian University, a sprinkling of uniforms. The law requires of every man in Prussia, without any exception, (unless of the princes royal) or excuse, unless of sickness, a year, or, if equipments and subsistence are provided by government, three years of actual service, and the student of medicine or theology has perhaps just come from the barracks or morning drill.

Two of the most largely attended cliniques, are those of Dr. Jaenchen at the Charité, and Dr. Dieffenbach at the University Hospital; the first an ophthalmic clinique, the last a general one, at which operations for tenotomy abound, in which Dr. D. is certainly very adroit.

One of the most interesting institutions of Berlin, is that for the instruction of the deaf and dumb, at which the new method is pursued of teaching them articulation. The success with which it is attended is certainly very astonishing, and, to an inexperienced observer like myself, quite satisfactory. The pupils converse with the instructers and with each other, so as to be intelligible to an ear as little practised as mine in the German language. The older ones can also read aloud and with a distinct enunciation, from an octavo volume of reading lessons, any passage that may be selected. But notwithstanding that by a patient imitation of the movements of the lips, tongue, larynx, and chest involved in articulation, this system seems to have achieved an impossibility; its expediency, as a general system of education for deaf mutes, is doubted by many practical observers, who say that the great length of time necessarily devoted by those who possess only ordinary imitative faculties, to the mere acquirement of an articulation, leaves not enough for more direct and important mental culture. A gentleman from New York is now here for the express purpose of investigating the methods and merits of the system, and his report will undoubtedly be of great value to those interested in the subject. But whether it be destined to supersede the older plan or not, it must be considered a very noticeable example of the ingenuity and perseverance of German teachers. This is, I believe, the largest institution of the sort in Germany; that at Liepsic, the oldest.

The schools for the instruction of the blind are here, and in the other cities which I have visited, less extensive than the one in Boston, which enjoys here a very high reputation. The education of the two blind mutes is spoken of in the strongest terms of admiration, and regarded as a much greater achievement than the teaching of the dumb to speak. Attached to the Deaf and Dumb Institute, is a class of idiots, in the instruction of whom great pains are taken, and a good deal accomplished.

You will not be surprised to learn, that just now, two new systems of medical practice are budding into favor in Germany. One is called the Traubencur (grape cure), and consists in living chiefly upon grapes, of which several pounds are to be eaten daily. I have not learned that its application is limited to any particular forms of disease, and am not able to give you the name of the originator of this happy discovery. It is now chiefly heard of in the regions of the Rhine, to the vineyards of which patients are, during the autumn, recommended to resort. The second system, at present less known, is entitled Aeropathy, and its paternity is claimed by a Dr. Flekeles of Prague, in Bohemia. The particulars are to be given in a book, which is forthcoming, but its essence is said to consist in alternate perspirations and exposures to currents of cold air. The first of these, the Traubencur, may be considered to be at present the *fashionable* one in Germany, but will probably soon yield to the other, of which the applicability is much more general, and the effects probably more decided.

On the route hither I passed a day at Halle, the medical school of which is a very good one, but chiefly observable from having in its possession the anatomical museum of the three Mechels. Some of the vast preparations by the elder Mechel are exceedingly elaborate, and, though more than a century old, in the most perfect condition. Connected with it is a very extensive collection in comparative anatomy, of more recent date. The anatomical prosector of the school claims to have made some discoveries in comparative anatomy, particularly of vertebrated animals. The only one which he pointed out to me, related to the difference in the form and arrangement of the spinous processes of the vertebræ, which he asserts, are found in every animal to be peculiarly and perfectly adapted to the movements and uses required of them. I have a faint idea, which is perhaps in the minds of some of your readers a vivid one, of having, a long time ago, on the other side of the water, read and listened to substantially the same statements. I intend shortly to leave for Vienna, and may write to you again. JOHN H. DIX.

